# Non-Orthogonal Random Access in MIMO Cognitive Radio Networks: Beamforming, Power Allocation, and Opportunistic Transmission

**DOI:** 10.1371/journal.pone.0169902

**Published:** 2017-01-11

**Authors:** Huifa Lin, Won-Yong Shin

**Affiliations:** 1 The Communications & Networking Laboratory, Dankook University, Yongin 448-701, Republic of Korea; 2 The Department of Computer Science and Engineering, Dankook University, Yongin 448-701, Republic of Korea; West Virginia University, UNITED STATES

## Abstract

We study secondary random access in multi-input multi-output cognitive radio networks, where a slotted ALOHA-type protocol and successive interference cancellation are used. We first introduce three types of transmit beamforming performed by secondary users, where multiple antennas are used to suppress the interference at the primary base station and/or to increase the received signal power at the secondary base station. Then, we show a simple decentralized power allocation along with the equivalent single-antenna conversion. To exploit the multiuser diversity gain, an opportunistic transmission protocol is proposed, where the secondary users generating less interference are opportunistically selected, resulting in a further reduction of the interference temperature. The proposed methods are validated via computer simulations. Numerical results show that increasing the number of transmit antennas can greatly reduce the interference temperature, while increasing the number of receive antennas leads to a reduction of the total transmit power. Optimal parameter values of the opportunistic transmission protocol are examined according to three types of beamforming and different antenna configurations, in terms of maximizing the cognitive transmission capacity. All the beamforming, decentralized power allocation, and opportunistic transmission protocol are performed by the secondary users in a decentralized manner, thus resulting in an easy implementation in practice.

## Introduction

As a promising technology to solve the fundamental problem of wireless spectrum scarcity, cognitive radio (CR) has been extensively investigated [1–4]. In spectrum sharing CR networks, secondary users (SUs) are allowed to transmit using the same frequency band assigned to primary users (PUs), provided that the requirement of an interference temperature constraint at the primary base station (PBS) is fulfilled [2]. The interference temperature is defined as a certain tolerable interference level at the primary receiver such as the PBS for uplink [5]. It is critical to protect the successful transmission of PUs by restricting the interference temperature caused by the secondary system [6–9].

On the one hand, since the number of user devices is likely to dramatically increase in the future, radio access networks are expected to support multiple access of massive users and achieve a higher spectrum efficiency [10–12]. Meanwhile, to avoid that the resulting overheads consume the scarce channel resource in CR networks, random access becomes an attractive option of multiple access due to the reduction of overheads for successful SU transmissions [13, 14]. On the other hand, successive interference cancellation (SIC) enables the receiver to decode multiple packets by successively subtracting the correctly decoded packets [15, 16], which helps to further improve the spectrum efficiency of random access. The principle of SIC has been first proposed in [17] and then applied to multi-input multi-output (MIMO) and code-division multiple access (CDMA) systems [18, 19].

### 0.1 Related Work

In MIMO CR networks, multiple antennas were exploited to improve the spectrum efficiency of the secondary system, while suppressing the interference to the primary system [20–25]. In [20], multi-antenna configuration was used to balance the interference reduction at the primary receivers and the spatial multiplexing in the secondary system. In [21], a beamforming method based on the game theory was proposed to maximize the spectrum efficiency of the secondary system. A beamforming scheme was also designed in terms of minimizing the transmit power of the secondary base station (SBS) [22]. In [23], a distributed beamforming design with limited multi-cell cooperation was proposed for CR networks. In [24], a joint design of admission control and beamforming was proposed to maximize the weighed sum rate of the secondary system with partial channel state information (CSI). In [25], joint beamforming, user scheduling, and power allocation strategies for a hierarchical cellular system were proposed to maximize the sum rate of the SUs by converting the original optimization problem into a convex semi-definite programming.

Different from the aforementioned approaches designed for centralized multiple access, for random access with an SIC-enabled receiver, decentralized power allocation (DPA) algorithms have received a great deal of attention [15, 16, 26–31]. Under the DPA algorithms, the transmit power is selected by each user independently, instead of being assigned by the BS. As originally pointed out in [26], DPA enhances the throughput of the random access system with multiple packet reception (MPR) capability. In [15], a random transmit power allocation scheme was proposed to achieve a higher throughput, assuming that the receiver is able to decode one packet from a collision. A DPA algorithm for the random access designed for the case that the SIC-enabled receiver decodes two packets from a collision was then proposed in [16], using the MPR model based on the signal-to-interference-and-noise ratio (SINR) measurement. For secondary random access *without* the SIC-enabled receiver in CR networks, the optimal threshold-based water-filling DPA policy was proposed in [28]. More recently, by modeling secondary random access as a multi-armed bandit problem, a fair channel-grouping scheme was proposed, while considering both the competition and the fairness between SUs [32]. In our previous studies [30, 31], we proposed DPA algorithms designed for a more general case that the SIC-enabled receiver is able to decode multiple packets for single-input single-output (SISO) random access systems.

Besides, there have been some related studies in a variaty of wireless networks [33–37]. In [33], the optimal power allocation and codeword rate design for secure relay communications were performed in Internet of Things (IoT) networks. For real-time positioning in millimeter wave D2D communications, a new threshold selection algorithm for energy detector-based ranging was proposed by employing a dynamic threshold based on an artificial neural network [34]. In [35], a new channel assignment method was proposed in wireless mesh networks. For cloud computing, a new resource allocation approach based on an imperfect information dynamic Stackelberg game using a hidden Markov model was proposed in [36]. In [37], a distributed and adaptive resource management approach was proposed in cloud-assisted CR vehicular networks. Moreover, under real and important application scenarios such as wireless clinical networks [14] and CR-based IoT networks [32], random access protocols were designed, which motivates us how to apply our proposed approaches in real applications.

### 0.2 Motivation and Contributions

In this paper, we study the secondary non-orthogonal random access in a MIMO CR network. Specially, we use a slotted ALOHA-type protocol and assume the SIC decoder that is able to resolve multiple received packets from one collision. For such a network, DPA is necessary since the received signal power of each transmitted packet needs to be properly distributed so that the SIC process performs better. At the same time, the interference temperature at the PBS caused by the secondary transmission also has to be controlled using the power allocation by the SUs. Moreover, the MIMO configuration can be exploited by using beamforming techniques to suppress the generated interference and/or to increase the received signal power at the SBS. In addition, the multiuser diversity gain in the network can also be exploited by opportunistically selecting SUs, thus yielding a further reduction of the total interference temperature at the PBS. In the random access system under consideration, all these methods including beamforming, DPA, and opportunistic transmission protocol (OTP) are conducted by each SU in a decentralized manner, based on its own local CSI at the transmitter (CSIT). Hence, our design paradigm is different from the well-investigated optimization methods for the centralized multiple access. To the best of our knowledge, it has not been studied in the literature to analyze the performance of secondary non-orthogonal random access in MIMO CR networks.

The primary focus in this work is on how to improve the sum rate performance of in the secondary *random access* system, while guaranteeing the successful reception of the primary system by confining the interference temperature at the PBS. A decentralized approach is adopted when the beamforming, DPA, and OTP are employed. It is worthy to highlight fundamental differences between our proposal and the existing approaches as follows.

- The conventional approaches [6–8, 20–25] designed for the multiple access channel or the point-to-point channel operate in a centralized manner, e.g., the power allocation is performed by the BS. As a result, the overheads required by the channel allocation stage consume the scarce spectrum resource in CR networks. On the contrary, in our proposal, the transmit power is randomly selected by each user in a decentralized manner, which is preferred by secondary random access in CR networks.
- In regards to the existing DPA approaches for CR networks, our proposal differs from [28] in the sense that the SIC-enabled receiver is adopted in the secondary system, which brings a potential sum rate improvement.
- Compared to our prior work designed for the single-antenna configuration [30, 31], the interference temperature at the PBS is further suppressed by using beamforming techniques.

The main contributions of this paper are summarized as follows.

- Three types of beamforming techniques are introduced for interference suppression at the PBS and/or the signal power enhancement at the SBS. More precisely, to increase the desired signal power, the beamforming type 1 (BF-1) is designed using the singular vector decomposition (SVD) of the channel matrix from each SU to the SBS. The beamforming type 2 (BF-2) is designed using the the SVD of the channel matrix to the PBS, so that the interference is suppressed. The beamforming type 3 (BF-3) is designed in terms of maximizing the signal-to-generated-interference ratio (SGINR) [38], which can be thought as a joint design based on both channel matrices from each SU to the SBS and the PBS.
- We use the DPA algorithm along with the equivalent single-antenna conversion, in the sense of maximizing the sum rate of secondary random access, under the constraint of the average interference temperature at the PBS.
- An OTP is proposed to exploit the multiuser diversity gain, leading to a further reduction of the total interference temperature at the PBS. In particular, before transmitting the packet, each SU calculates its generating interference independently. Taking this value as a metric, the SU compares it to a threshold *η* sent by the SBS. If the resulting interference of an SU is less than *η*, which means that relatively smaller interference is generated, then the SU becomes a transmitter candidate and allocates a power level according to DPA. The OTP is efficiently combined with the beamforming and DPA at the transmitter side.
- The average interference temperature at the PBS is analyzed and then validated via computer simulations. For three types of beamforming techniques and various antenna configurations, we examine the optimal value of *η* according to different quality of service (QoS) targets, while maximizing the net system performance, named cognitive transmission capacity (CTC), which is used to measure the spectrum efficiency achieved by the secondary system under the condition that the reception at the PBS is protected. In practice, both the DPA procedure and the optimal parameter decision of the OTP are made first by the SBS offline. Thereafter, the optimal parameter values of the OTP are broadcast over the network. Each SU performs the beamforming, DPA, and OTP independently, without any further control signalling from the SBS. Since our decentralized approaches require only a small amount of control signalling from the BS, they are suitable for random access scenarios where it is difficult or expensive to establish the centralized control (e.g., random access of SUs in CR-based IoT networks [32]).
- Moreover, we also evaluate the performance of our proposal under a more practical standardized model, by taking into account the geometric distribution of nodes and the path loss component of wireless links. Numerical results confirm that our approaches under the practical setting lead to a similar sum rate trend to that obtained under a relatively ideal setting. In addition, it is found that the coverage of the secondary system and the distance between the PBS and SBS heavily affect the resulting interference temperature at the PBS. Optimal parameter values of the OTP are numerically found in terms of maximizing the CTC, which also depends on the geometric distribution in our practical model.

This paper is organized as follows. Section 1 shows the system model. Section 2 presents the transmit beamforming techniques, the DPA algorithm, and the OTP. Section 3 shows not only the analysis on the average interference temperature but also the computational complexity of the proposed approaches. Section 4 provides numerical results for performance validation. Section 5 concludes this paper.

*Notations:* C denotes the field of complex numbers; (⋅)^*H*^ indicates the conjugate transpose and; ‖⋅‖^2^ denotes the *L*_2_-norm.

## 1 System and Channel Models

**Fig 1:**
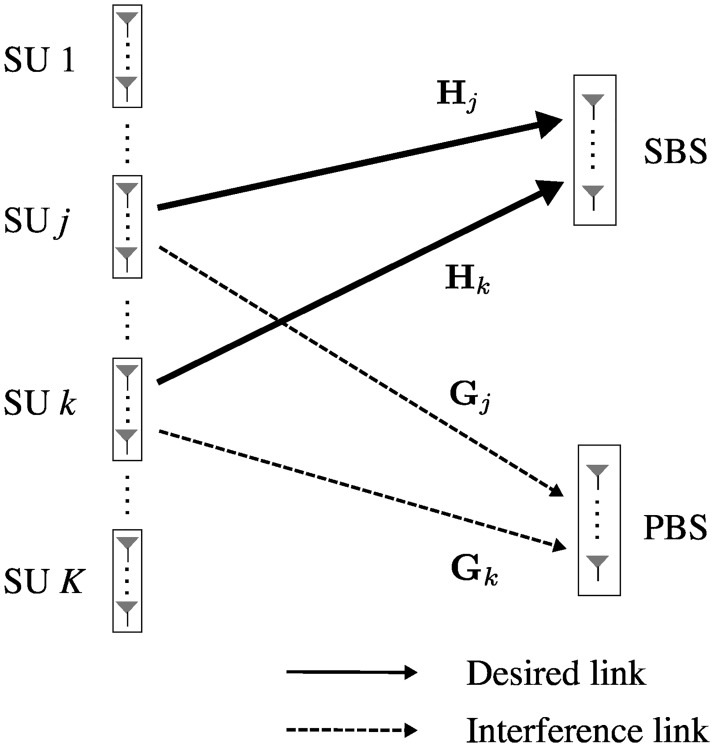
The system model. The secondary random access in our MIMO CR network, consisting of *K* SUs, one SBS, and one PBS.

As shown in Fig 1, our CR network consists of *K* SUs with *N*_*t*_ antennas each, one SBS with *N*_*r*_ antennas, and one PBS with *N*_*r*_ antennas. We focus on secondary random access from the SUs to the SBS. A slotted ALOHA-type protocol is adopted and full-loaded traffic in the network is assumed. Channel coefficient matrices from the *k*-th SU to the SBS and the PBS are denoted by $H_{k}\in C^{N_{r}\times N_{t}}$ and $G_{k}\in C^{N_{r}\times N_{t}}$, respectively, where *k* ∈ {1, ⋯, *K*}. It is assumed that full CSI is available at the SBS but each SU has only the local CSI of its own transmit link to the PBS and/or the SBS. The CSI at the transmitter can be acquired by exploiting the channel reciprocity if time division duplex (TDD) mode is adopted.

At the transmitter side, it is assumed that the information blocks are protected from the noise and interference by strong channel codes and the bit-interleaved coded modulation (BICM) [39]. The channel coding and the modulation together constitute the base code with the data rate *R*_*o*_. Let *x*_*k*_ denote the symbol after the channel coding and modulation, where the symbol power is normalized to $E[|x_{k}{|}^{2}]=1$. Then, transmit beamforming is performed by multiplying a precoding vector $w_{k}\in C^{N_{t}\times 1}$ (to be specified in Section 2). Let *e*_*k*_ denote the transmit power of the *k*-th SU. Then, the signal of the *k*-th SU is given by $\sqrt{e_{k}}w_{k}x_{k}$.

The received signal at the SBS is given by

$$\begin{array}{c} y=\sum_{i=1}^{K}H_{i}\sqrt{e_{i}}w_{i}x_{i}+n, \end{array}$$ (1)

where each entry of $n\in C^{N_{r}\times 1}$ is modeled as a complex Gaussian random variable following $\text{CN}(0,N_{0})$. Similarly as in [40], the SBS is able to successfully decode the *k*-th SU’s packet if the SINR exceeds a *decoding threshold* given by $\rho =(2^{R_{o}}-1)\left(1+\Delta \right)$. Here, Δ ≥ 0 is a decoding threshold margin that indicates the performance gap incurred by a practical code design with respect to the ideal channel codes. Accordingly, it is examined whether a packet can be successfully decoded by comparing its SINR with *ρ*, instead of using an explicit code design. Upon the successfully decoding, the SIC process reconstructs and subtracts the signal of the decoded packet from the received signal. The SIC process is repeated until all packets are decoded except that none’s SINR exceeds the decoding threshold.

In our CR network, the SUs share the uplink frequency band with the primary system. To guarantee successful reception at the PBS, we need to control the average interference temperature at the PBS such that $E\left[\sum_{k=1}^{K}∥G_{k}w_{k}{∥}^{2}e_{k}\right]\leq E_{\text{IT}}$, where *E*_IT_ > 0 is a certain constant representing the tolerable interference level at the PBS. In addition, since each SU independently selects its transmit power level under the fading environment, the actual interference temperature for a certain time slot may exceed *E*_IT_, thus causing an outage event with a probability of $P_{\text{out}}=\mathrm{Pr}(\sum_{k=1}^{K}∥G_{k}w_{k}{∥}^{2}e_{k}>E_{\text{IT}})$.

## 2 Proposed Methods

For secondary random access with the SIC-enabled receiver in our MIMO CR network, it is crucial to control or even further reduce the interference temperature at the PBS, while enhancing the sum rate performance of the secondary system. In this section, we introduce several methods that operate in a decentralized manner to achieve these targets.

### 2.1 Overall Procedure

**Fig 2:**
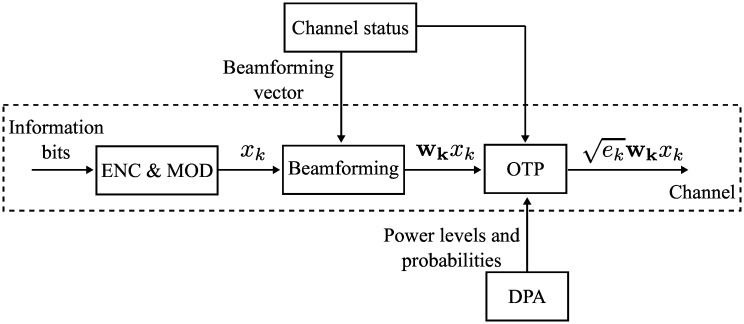
The diagram of the overall system. The overall procedure at the *k*-th SU, where **G**_*k*_ is the channel matrix to the PBS, **H**_*k*_ is the channel matrix to the SBS, **w**_*k*_ is the precoding vector, *e*_*k*_ is the transmit power, and **E**, **p** are the discrete power levels and the corresponding probabilities, respectively.

As illustrated in Fig 2, an overall procedure for an arbitrary SU is explained as follows. First, the information bits are encoded and modulated into complex symbol *x*_*k*_ as described in the previous section. Then, we employ three different types of beamforming techniques by finding a proper precoding vector **w**_**k**_. The precoding vector **w**_**k**_ is designed in the sense of suppressing the generating interference to the PBS and/or enhancing the desired signal power at the PBS. Afterwards, the OTP is employed, where each SU decides whether to transmit or not by comparing its channel gain to the SBS with a certain threshold. If the *k*-th SU decides to transmit, then it randomly selects a power level *e*_*k*_ based on the sets of power levels and probabilities generated by the DPA algorithm. Finally, the transmitted signal is expressed as $\sqrt{e_{k}}w_{k}x_{k}$.

We employ a modular design concept, where different functions are encapsulated in independent blocks. There are pros and cons by using this design concept. The modular design can be simply implemented and is analytically tractable, compared to the joint optimization. Another advantage comes from the fact that individual function blocks could be further improved without any complex modification of the overall structure. In the following subsections, the details of each function block are described.

### 2.2 Beamforming

To suppress the generating interference to the PBS and/or to enhance the received signal power at the SBS through transmit beamforming, each SU needs to find a proper precoding vector $w_{k}\in C^{N_{t}\times 1}$. For the *k*-th SU, the generating interference is given by ‖**G**_*k*_**w**_*k*_‖^2^ *e*_*k*_, where the transmit power *e*_*k*_ is determined by using DPA (to be specified later). In our work, we assume that each SU transmits only one data stream over multiple antennas, which is sufficient to achieve the full multiplexing gain under our random access model.

#### 2.2.1 Beamforming Type 1 (BF-1)

Suppose that only the channel matrix to the SBS is available at each transmitter (SU). Then, we are able to provide a straightforward design by obtaining the precoding vector **w**_S,*k*_ based on the SVD of the channel matrix from the *k*-th SU to the SBS. The SVD of **H**_*k*_ is given by

$$H_{k}=U_{\text{S},k}\Sigma_{\text{S},k}V_{\text{S},k}^{H},$$ (2)

where $U_{\text{S},k}\in C^{N_{r}\times N_{r}}$ and $V_{\text{S},k}\in C^{N_{t}\times N_{t}}$ are the unitary matrices, $\Sigma_{\text{S},k}=\text{diag}\;(\sigma_{\text{S},k}^{\left[1\right]},\cdots ,\sigma_{\text{S},k}^{\left[N_{t}\right]})$, and $\sigma_{\text{S},k}^{\left[1\right]}\geq \cdots \geq \sigma_{\text{S},k}^{\left[N_{t}\right]}$. The precoding vector $w_{\text{S},k}={\left[V_{\text{S},k}^{1,1},\cdots ,V_{\text{S},k}^{N_{t},1}\right]}^{T}$ is given by the first column of **V**_S,*k*_, corresponding to the largest singular value $\sigma_{\text{S},k}^{\text{max}}=\sigma_{\text{S},k}^{\left[1\right]}$. Here, $V_{\text{S},k}^{n,1}$ denote the (*n*, 1)-th element of **V**_S,*k*_. The transmit signal of the *k*-th SU is then given by $\sqrt{e_{k}}w_{\text{S},k}x_{k}$. After post-processing, we have

$$u_{\text{S},k}^{H}H_{k}\sqrt{e_{k}}w_{\text{S},k}x_{k}=\sqrt{e_{k}}\sigma_{\text{S},k}^{\left[1\right]}x_{k},$$ (3)

where the receive shaping vector **u**_S,*k*_ is given by the first column of **U**_S,*k*_.

The SIC process is employed to decode multiple received packets, which are assumed to be ordered according to the received power $e_{\pi (1)}{\left(\sigma_{\text{S},\pi (1)}^{\left[1\right]}\right)}^{2}\geq e_{\pi (2)}{\left(\sigma_{\text{S},\pi (2)}^{\left[1\right]}\right)}^{2}\geq \cdots \geq e_{\pi (\tilde{K})}{\left(\sigma_{\text{S},\pi (\tilde{K})}^{\left[1\right]}\right)}^{2}$, where $\tilde{K}$ is the number of SUs with positive transmit power. For instance, to decode the packet of the *π*(1)-th SU from the received signal

$$\begin{array}{c} y_{\pi (1)}=H_{\pi (1)}\sqrt{e_{\pi (1)}}w_{\text{S},\pi (1)}x_{\pi (1)}+\sum_{i=2}^{\tilde{K}}H_{\pi (i)}\sqrt{e_{\pi (i)}}w_{\text{S},\pi (i)}x_{\pi (i)}+n, \end{array}$$ (4)

multiplying **y**_*π*(1)_ by $u_{\text{S},k}^{H}$ leads to

$$\begin{array}{cc} u_{\text{S},k}^{H}y_{\pi (1)} & =u_{\text{S},k}^{H}H_{\pi (1)}\sqrt{e_{\pi (1)}}w_{\text{S},\pi (1)}x_{\pi (1)}+u_{\text{S},k}^{H}\left(\sum_{i=2}^{\tilde{K}}H_{\pi (i)}\sqrt{e_{\pi (i)}}w_{\text{S},\pi (i)}x_{\pi (i)}+n\right)\\ & =\sqrt{e_{\pi (1)}}\sigma_{\text{S},\pi (1)}^{\left[1\right]}x_{\pi (1)}+u_{\text{S},k}^{H}\left(\sum_{i=2}^{\tilde{K}}H_{\pi (i)}\sqrt{e_{\pi (i)}}w_{\text{S},\pi (i)}x_{\pi (i)}+n\right), \end{array}$$ (5)

which results in a simple scalar form. Here, the SINR of the target signal is given by

$${\text{SINR}}_{\pi (1)}=\frac{e_{\pi (1)}{\left(\sigma_{\text{S},\pi (1)}^{\left[1\right]}\right)}^{2}}{\sum_{i=2}^{\tilde{K}}e_{\pi (i)}{\left|u_{\text{S},\pi (i)}^{H}H_{\pi (i)}w_{\text{S},\pi (i)}\right|}^{2}+N_{0}}.$$ (6)

If *x*_*π*(1)_ is successfully decoded, then its signal is reconstructed and subtracted from **y**_*π*(1)_. Afterwards, we have

$$y_{\pi (2)}=\sum_{i=2}^{\tilde{K}}H_{\pi (i)}\sqrt{e_{\pi (i)}}w_{\text{S},\pi (i)}x_{\pi (i)}+n.$$ (7)

The receiver continues on decoding *x*_*π*(2)_ from **y**_*π*(2)_. This process is repeated until all packets are successfully decoded except that no packet’s SINR exceeds the decoding threshold.

#### 2.2.2 Beamforming Type 2 (BF-2)

An alternative beamforming method is to use the precoding vector **w**_P,*k*_ obtained from the SVD of **G**_*k*_, which is given by

$$G_{k}=U_{\text{P},k}\Sigma_{\text{P},k}V_{\text{P},k}^{H},$$ (8)

where $U_{\text{P},k}\in C^{N_{r}\times N_{r}}$ and $V_{\text{P},k}\in C^{N_{r}\times N_{r}}$ are the unitary matrices. Note that the CSIT to the PBS at each SU is assumed. To minimize the generating interference, the precoding vector is given by

$$\begin{array}{cc} w_{\text{P},k} & =\underset{v_{\text{P},k}}{\mathrm{argmin}}∥G_{k}v_{\text{P},k}{∥}^{2}=v_{\text{P},k}^{\left[N_{t}\right]}\\ & \text{s.t.}\;∥v_{\text{P},k}{∥}^{2}=1, \end{array}$$ (9)

where $v_{\text{P},k}^{\left[N_{t}\right]}$ is the *N*_*t*_-th column of **V**_P,*k*_, which follows the similar steps to those in [41].

#### 2.2.3 Beamforming Type 3 (BF-3)

Furthermore, in order to jointly enhance the received signal power at the SBS and reduce the interference power at the PBS, each SU can perform beamforming in the sense of maximizing the SGINR. In this case, the precoding vector **w**_J,*k*_ is given by

$$\begin{array}{cc} w_{\text{J},k} & =\underset{v_{\text{J},k}}{\mathrm{argmax}}\frac{∥H_{k}v_{\text{J},k}{∥}^{2}}{1+∥G_{k}v_{\text{J},k}{∥}^{2}}\\ & =\underset{v_{\text{J},k}}{\mathrm{argmax}}\frac{v_{\text{J},k}^{H}\left(H_{k}^{H}H_{k}\right)\;v_{\text{J},k}}{v_{\text{J},k}^{H}\left(I_{N_{t}}+G_{k}^{H}G_{k}\right)\;v_{\text{J},k}}\\ & \text{s.t.}\;∥v_{\text{J},k}{∥}^{2}=1, \end{array}$$ (10)

which can be solved using the generalized eigenproblem and the Rayleigh-Ritz theorem [38]. To obtain this precoding vector, we first define the SGINR-based covariance matrix as

$$J_{k}={\left(I_{N_{t}}+G_{k}^{H}G_{k}\right)}^{-1}\left(H_{k}^{H}H_{k}\right).$$ (11)

Then, the eigenvalue decomposition of **J**_*k*_ is given by

$$J_{k}=V_{\text{SGINR},k}\Sigma_{\text{SGINR},k}V_{\text{SGINR},k}^{H},$$ (12)

where $V_{\text{SGINR},k}\in C^{N_{r}\times N_{r}}$ is the unitary matrix and **Σ**_SGINR,*k*_ is the diagonal matrix whose elements are the eigenvalues. The precoding matrix **w**_J,*k*_ maximizing the SGINR is given by the first column of **V**_SGINR,*k*_, corresponding to the largest eigenvector. Note that to perform the eigenvalue decomposition, the CSIT to both the SBS and the PBS is assumed to be available.

For the BF-2 and BF-3 cases, we employ the minimum mean-square error (MMSE)-SIC detection method [42]. Let $\tilde{H}=\left[h_{\pi (1)},\cdots ,h_{\pi (\tilde{K})}\right]\in C^{N_{r}\times \tilde{K}}$ denote the equivalent overall channel matrix after ordering, e.g., the *i*-th column is given by $h_{\pi (i)}=H_{\pi (i)}w_{\pi (i)}\in C^{1\times N_{r}}$. The receiver first performs the MMSE filtering for the *π*(1)-th SU’s signal, which results in

$${\tilde{y}}_{\pi (1)}={\left({\tilde{H}}_{\pi (1)}^{H}{\tilde{H}}_{\pi (1)}+\frac{N_{0}}{e_{\pi (1)}}I_{N_{r}}\right)}^{-1}{\tilde{H}}_{\pi (1)}^{H}y,$$ (13)

where ${\tilde{H}}_{\pi (1)}=\tilde{H}$. If this signal is successfully decoded, then it is subtracted from the received signal and the remaining signal is given by

$$y_{\pi (2)}=\sum_{i=2}^{\tilde{K}}\sqrt{e_{\pi (i)}}H_{\pi (i)}w_{\pi (i)}x_{\pi (i)}+n.$$ (14)

Accordingly, the first column of the overall channel matrix is removed and we have ${\tilde{H}}_{\pi (2)}=\left[h_{\pi (2)},\cdots ,h_{\pi (\tilde{K})}\right]\in C^{N_{r}\times \left(\tilde{K}-1\right)}$. The receiver continues on performing the MMSE-based decoding of the next SU’s signal along with ${\tilde{H}}_{\pi (2)}$ and **y**_*π*(2)_. Note that since **w**_*k*_ is obtained from **G**_*k*_ that is not known at the SBS, the SUs need to deliver this information to the SBS via the uplink channel.

### 2.3 Decentralized Power Allocation (DPA)

The DPA algorithm was originally introduced for the SISO Gaussian channel to maximize the sum rate of random access with the SIC-capable receiver in our previous work [30], and was later extended to the secondary random access scenario in a SISO CR network [31]. In the algorithm, during each time slot, each SU randomly selects its transmit power from a set of discrete power levels **E** = {*E*_0_, ⋯, *E*_*L*_} according to the probabilities **p** = [*p*_0_, ⋯, *p*_*L*_], where *L* is the number of power levels. The power levels and the corresponding probabilities are obtained for the Gaussian channel according to the given noise power *N*_0_. Note that *E*_0_ = 0 indicates the idle status and *E*_*L*_ ≤ 1 is assumed due to the maximum transmit power constraint. The probability that the *k*-th SU selects *E*_*l*_ is given by *p*_*l*_ = Pr(*e*_*k*_ = *E*_*l*_). Now, we describe the DPA algorithm for the MIMO case with the equivalent single-antenna conversion. The received signal of the *k*-th SU at the SBS is given by $H_{k}\sqrt{e_{k}}w_{k}x_{k}$, which corresponds to a randomly selected power level from **E**, i.e., $∥H_{k}w_{k}\sqrt{e_{k}}{∥}^{2}=E_{l}$ for *l* ∈ {1, ⋯, *L*}. Thus, the transmit power is given by $e_{k}=\frac{E_{l}}{∥H_{k}w_{k}{∥}^{2}}$.

In what follows, the DPA algorithm for the equivalent SISO channel is briefly described. Let *T*_*l*_ denote the MAC throughput provided by the *l*-th power level assuming that the packets of higher power levels are subtracted. Let *ζ*_*i*_ denote the probability that the SIC process successfully continues to a lower power level than the *l*-th power level. Then, the throughput optimization problem used to find both **E** and **p** is formulated as follows.

$$\underset{E,p}{\text{maximize}}\;T_{L}+\sum_{j=1}^{L-1}\left(T_{j}\prod_{i=j+1}^{L}\zeta_{i}\right)$$ (15a)

$$\text{subject}\;\text{to}\;0<E_{i}\leq 1,E_{i-1}-E_{i}<0,\;i=1,\ldots ,L$$ (15b)

$$0<p_{i}<1,\sum_{i=1}^{L}p_{i}\leq 1,\;i=1,\ldots ,L$$ (15c)

$$\sum_{i=1}^{L}Kp_{i}E_{i}\leq E_{\text{sum}},$$ (15d)

where *E*_sum_ is the constraint of the sum transmit power. The objective function of this optimization problem in Eq (15a) is the overall throughput of the secondary system. Since this optimization problem is almost intractable due to the complicated objective function, we introduce a bottom-up per-level iterative algorithm to obtain **E** and **p**. The idea behind is to convert the overall optimization problem into simple tractable per-level sub-problems that are proved to be quasi-concave. The iterative process is used to obtain the power and the probability of the current level, based on the information of lower levels. For more details, we refer to [30].

### 2.4 Opportunistic Transmission Protocol (OTP)

We propose the OTP to further reduce the interference temperature at the PBS by exploiting the multiuser diversity gain, where only the SUs generating relatively less interference transmit. A metric

$$\mu ≜∥G_{k}w_{k}{∥}^{2}$$ (16)

that represents the generating interference from the *k*-th SU to the PBS is used to divide all the SUs into two groups. The SUs with *μ* ≤ *η* belong to the candidate group in which the transmit power is allocated, where *η* is a given threshold. The probability that a certain SU is in the candidate group is then given by *δ* = Pr(*μ* ≤ *η*). The other SUs with *μ* > *η* belong to the idle group that will not transmit in the current time slot.

We need to adjust the probabilities **p** when the OTP is adopted. Let $p_{l}^{\prime }\geq p_{l}$ denote the adjusted probability for *E*_*l*_, which can be obtained by establishing the following theorem.

**Theorem 1** *Suppose that the OTP is used for secondary random access in our MIMO CR network. The probabilities corresponding to the transmit power levels* **E** *are adjusted as*

$$\left\{\begin{array}{cccc} & p_{0}^{\prime }=\frac{\left(p_{0}-\left(1-\delta \right)\right)}{\delta },\;p_{j}^{\prime }=\frac{p_{j}}{\delta },\; & & \text{if}\;\delta \geq 1-p_{0},\\ & p_{0}^{\prime }=0,\;p_{j}^{\prime }=\frac{p_{j}}{\left(1-p_{0}\right)},\; & & \text{if}\;\delta <1-p_{0}, \end{array}\right.$$ (17)

*where* *j* ∈ {1, ⋯, *L*}.

*Proof:* For *δ* ≥ 1 − *p*_0_, the overall idle SUs consist of the SUs in the idle group and the SUs in the candidate group with the allocated power level *E*_0_. Thus, we have $p_{0}=\left(1-\delta \right)+p_{0}^{\prime }\delta$. Since the adjusted probabilities of non-zero power levels are scaled at the same time, it follows that $p_{j}=p_{j}^{\prime }\delta ,\;\forall j\in \left\{1,\cdots ,L\right\}$. By solving these equations, we obtain the adjusted probabilities for *δ* ≥ 1 − *p*_0_. For *δ* < 1 − *p*_0_, all the SUs in the candidate group should transmit. Thus, we have $p_{0}^{\prime }=0$ and $\sum_{j=1}^{L}p_{j}^{\prime }=\sum_{j=1}^{L}\sigma p_{j}=1$, where *σ* is a scaling factor. By solving these equations, we obtain the adjusted probabilities for *δ* < 1 − *p*_0_, which completes the proof of this theorem.

It is possible to obtain the ratio of the candidate SUs, *δ*, according to the cumulative distribution function (CDF) of *μ*. In particular, for the BF-1 case, the precoding vector **w**_*k*_ is a column of the unitary matrix that is independent of **G**_*k*_. Thus, *μ* follows the *χ*^2^ distribution with *N*_*r*_ degrees of freedom, which is given by

$$\mathrm{Pr}\left(\mu \leq \eta \right)=\frac{\gamma \left(\frac{N_{r}}{2},\frac{\eta }{2}\right)}{\Gamma \left(\frac{N_{r}}{2}\right)},$$ (18)

where *γ*(⋅, ⋅) is the lower incomplete Gamma function and Γ(⋅) is the Gamma function. For the other two beamforming techniques BF-2 and BF-3, since the precoding vectors are not independent of **G**_*k*_, the corresponding CDFs cannot be given by Eq (18), but still can be obtained by offline numerical methods. This known value of *δ* is then used to obtain the probabilities $\left\{p_{0}^{\prime },\cdots ,p_{L}^{\prime }\right\}$ from Theorem 1. The value of *η* is broadcast over the network. Each SU checks whether it is in the candidate group by comparing its *μ* with *η*. If the *k*-th SU belongs to the candidate group, then it randomly selects its power level (e.g., *E*_*l*_) from **E** according to the set of adjusted probabilities $\left\{p_{0}^{\prime },\cdots ,p_{L}^{\prime }\right\}$.

**Fig 3:**
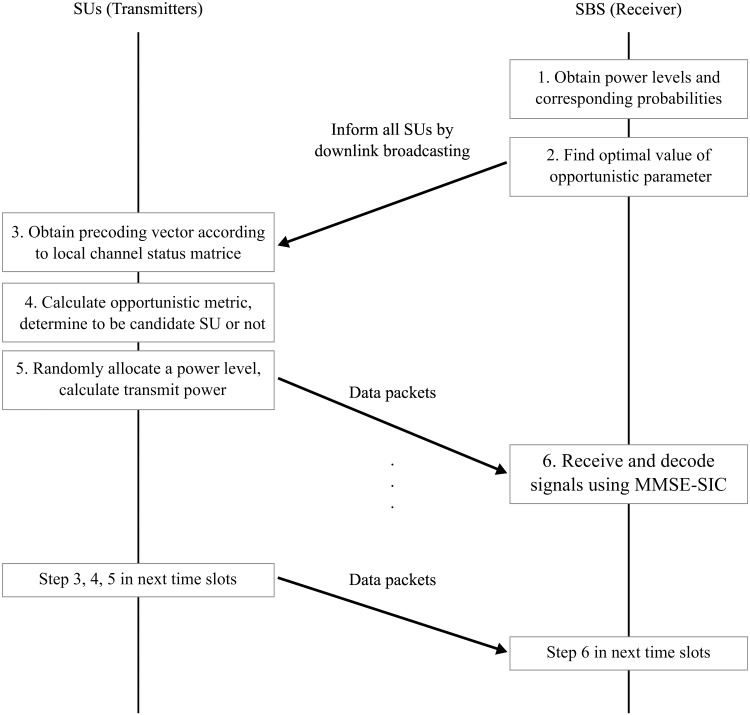
The diagram of the overall communication flow. The communication flow between the SUs and the SBS using our proposed approaches.

The overall procedure is illustrated in Fig 3. The SBS first obtains **E** and **p** using DPA and then finds the optimal *η* value offline. These parameters including **E**, **p**, and *η* are broadcast over the network. Each SU obtains the precoding vector, calculates the metric *μ* in Eq (16), and determines whether to belong to the candidate group, according to its local CSI. Each candidate SU randomly selects a power level from **E**, adjusts the transmit power according to the channel gain, and finally transmits its packet after the precoding. At the receiver end, the SBS receives and decodes the packets using the aforementioned MMSE-SIC detection method in Section 2.2.3.

## 3 Discussion

### 3.1 Analysis on Interference Temperature

In this subsection, we analyze the average interference temperature at the PBS by establishing the following theorem.

**Theorem 2** *Suppose that the OTP is used for secondary random access in our MIMO CR network, where only the SUs with* *μ* ≤ *η* *belong to the candidate group. Then, the average interference temperature is expressed as*

$$\begin{array}{c} E\;\left[\sum_{i=1}^{K}e_{i}{∥G_{i}w_{i}∥}^{2}\right]=K\cdot \sum_{l=1}^{L}E_{l}p_{l}^{\prime }\cdot \int_{0}^{\eta }\frac{\mu f(\mu )}{∥H_{k}w_{k}{∥}^{2}}d\mu , \end{array}$$ (19)

*where* *μ* = ‖**G**_*k*_ **w**_*k*_‖^2^ *and* *f*(*μ*) *is the probability density function (PDF) of* *μ*.

*Proof:* The average interference temperature caused by an arbitrary SU (e.g., the *k*-th SU) is given by

$$\begin{array}{c} E\;\left[e_{k}{∥G_{k}w_{k}∥}^{2}\right]. \end{array}$$ (20)

Let us denote ${\tilde{e}}_{k}=e_{k}{∥H_{k}w_{k}∥}^{2}\in E$ through the DPA algorithm for the equivalent SISO channel. By taking into account the OTP, the expectation of the interference temperature is given by

$$\begin{array}{c} K\cdot \int_{0}^{\eta }\frac{{\tilde{e}}_{k}}{∥H_{k}w_{k}{∥}^{2}}\mu f\left(\mu \right)d\mu , \end{array}$$ (21)

where *μ* = ‖**G**_*k*_ **w**_*k*_‖^2^ ≥ 0. The integral is taken over *μ* ∈ [0, *η*] because only the SUs with *μ* ≤ *η* have an opportunity to transmit. Since ${\tilde{e}}_{k}$ is randomly determined by the DPA, independent of the channel status, Eq (21) can be rewritten as

$$\begin{array}{cc} & K\cdot E\left[{\tilde{e}}_{k}\right]\cdot \int_{0}^{\eta }\frac{\mu f(\mu )}{∥H_{k}w_{k}{∥}^{2}}d\mu \\ & =K\cdot \sum_{l=1}^{L}E_{l}p_{l}^{\prime }\cdot \int_{0}^{\eta }\frac{\mu f(\mu )}{∥H_{k}w_{k}{∥}^{2}}d\mu , \end{array}$$ (22)

where *E*_*l*_ and $p_{l}^{\prime }$ indicate the power and the corresponding probability of the *l*-th power level, respectively. This completes the proof of this theorem.

From this theorem, it is seen that the total average interference temperature at the PBS depends on both the expectation of the generated interference from each individual SU and the number of SUs. Moreover, the analysis can be simplified by separating the item $\sum_{l=1}^{L}E_{l}p_{l}^{\prime }$ from the integral, which is determined by the DPA. Thus, we focus on how the beamforming techniques and the OTP affect the average interference temperature.

In particular, for the BF-2 case, the precoding vector **w**_*k*_ is independent of **H**_*k*_ since it is obtained from the SVD of **G**_*k*_. Hence, ‖**H**_*k*_ **w**_*k*_‖^2^ follows the *χ*^2^ distribution with *N*_*r*_ degrees of freedom, which thus results in the following average interference temperature:

$$\begin{array}{cc} & \frac{K}{E\;\left[∥H_{k}w_{k}{∥}^{2}\right]}\cdot \sum_{l=1}^{L}E_{l}p_{l}^{\prime }\cdot \int_{0}^{\eta }\mu f(\mu )d\mu \\ & =\frac{K}{N_{r}}\cdot \sum_{l=1}^{L}E_{l}p_{l}^{\prime }\cdot \int_{0}^{\eta }\mu f(\mu )d\mu . \end{array}$$ (23)

Here, $\int_{0}^{\eta }\;\mu f(\mu )d\mu$ can be computed by the known PDF of *μ*. However, for the other two beamforming techniques BF-1 and BF-3, since **w**_*k*_ is not independent of **H**_*k*_, it is not straightforward to obtain such an explicit form from Theorem 2.

For the case without the OTP, all the SUs have an equal opportunity to transmit, regardless of the channel status. Then, Eq (20) can be expressed as

$$\begin{array}{cc} K\cdot E[e_{k}∥G_{k}w_{k}{∥}^{2}] & =K\cdot E\;\left[\frac{{\tilde{e}}_{k}}{∥H_{k}w_{k}{∥}^{2}}{∥G_{k}w_{k}∥}^{2}\right]\\ & =K\cdot E\left[{\tilde{e}}_{k}\right]E\;\left[\frac{∥G_{k}w_{k}{∥}^{2}}{∥H_{k}w_{k}{∥}^{2}}\right]\\ & =K\cdot \sum_{l=1}^{L}E_{l}p_{l}\cdot E\;\left[\frac{∥G_{k}w_{k}{∥}^{2}}{∥H_{k}w_{k}{∥}^{2}}\right], \end{array}$$ (24)

where $\sum_{l=1}^{L}E_{l}p_{l}$ can be easily computed since **E** and **p** are given by the DPA. The term $E\;[\frac{∥G_{k}w_{k}{∥}^{2}}{∥H_{k}w_{k}{∥}^{2}}]$ depends only on the channel status and can be numerically obtained by the SBS offline. Hence, it is possible for the SBS to estimate the average interference temperature by using the given DPA algorithm, beamforming techniques, and the distribution of channel gains.

### 3.2 Complexity of the Proposed Methods

In this subsection, we analyze the computational complexity of the proposed approaches including the beamforming, DPA, and OTP by examining the required floating operations (flops) [43]. We adopt the big O notation to capture the asymptotic performance as *N*_*t*_ and *N*_*r*_ are sufficiently large, where *f*(*x*) = *O*(*g*(*x*)) means that there exist constants *C* and *c* such that *f*(*x*) ≤ *Cg*(*x*) for all *x* > *c*. With respect to the beamforming techniques, the BF-1 and BF-2 cases need to perform SVD of the channel matrix (**H**_*k*_ or **G**_*k*_) to obtain the precoding vectors, and thus the complexity is given by $O(N_{r}N_{t}^{2})$ [43]. For the BF-3 case, since it is required to first obtain the covariance matrix **J**_*k*_ and then perform the eigenvalue decomposition of **J**_*k*_, the overall complexity is given by $O(N_{t}^{3}+N_{r}N_{t}^{2})$ [43]. Unlike the beamforming techniques, we obtain the parameter values of the DPA and OTP offline and broadcast them over the network. Note that these parameter values keep the same regardless of a given channel realization (e.g., the average SNR and the fading distribution). Thus, at the transmitter end, the complexity of the DPA and OTP arises only from computing ‖**H**_*k*_**w**_*k*_‖^2^ or ‖**G**_*k*_**w**_*k*_‖^2^, which is given by *O*(*N*_*r*_ *N*_*t*_). The complexity of the proposed methods is summarized in Table 1.

**Table 1:** The computational complexity of the proposed approaches.

Method		Computational complexity
Beamforming	BF-1	$O(N_{r}N_{t}^{2})$
	BF-2	$O(N_{r}N_{t}^{2})$
	BF-3	$O(N_{t}^{3}+N_{r}N_{t}^{2})$
DPA		*O*(*N*_*r*_*N*_*t*_)
OTP		*O*(*N*_*r*_*N*_*t*_)

## 4 Numerical Results

We evaluate the performance of the proposed methods via intensive computer simulations. The simulation environments are described as follows. We adopt a slotted ALOHA-type random access protocol in the MIMO CR network with *K* = 100 full-loaded SUs contending for transmission to the SBS, without using the carrier sensing or the backoff mechanism. The transmit power levels **E** and the corresponding probabilities **p** are obtained according to the DPA algorithm in [31], where the noise power density, *N*_0_, is assumed to be 10^−2^.

### 4.1 Sum Rate and Energy Efficiency

**Fig 4:**
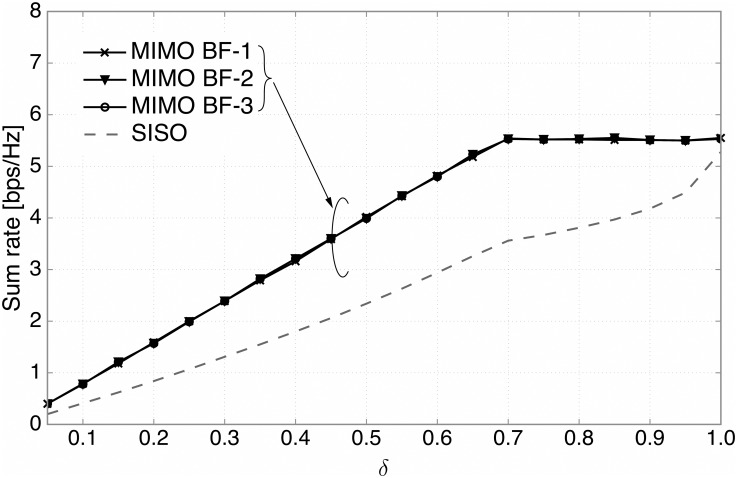
The sum rate performance. The sum rate versus the ratio of candidate SUs, *δ*, for three types of beamforming techniques (*N*_*t*_ = 2, *N*_*r*_ = 4) and the SISO case.

Fig 4 shows the sum rate performance of our secondary system versus *δ* for both the MIMO (*N*_*t*_ = 2, *N*_*r*_ = 4) and SISO configurations using the proposed methods, where *δ* is the ratio of the candidate SUs. Compared to the SISO case, employing one of three beamforming techniques provides superior sum rate performance for all ranges of *δ*. For each beamforming type, it is observed that the sum rate first increases with *δ* when *δ* is smaller than a certain value and then gets saturated. This is because the OTP controls the number of candidate SUs. To be specific, for small *δ*, there are not a sufficient number of candidate SUs for transmission. Thus, as *δ* increases, more SUs tend to be in the candidate group and the sum rate increases accordingly. For large *δ*, there are a sufficient number of candidate SUs. In this case, the sum rate performance is independent of *δ* and depends only on the DPA itself.

**Fig 5:**
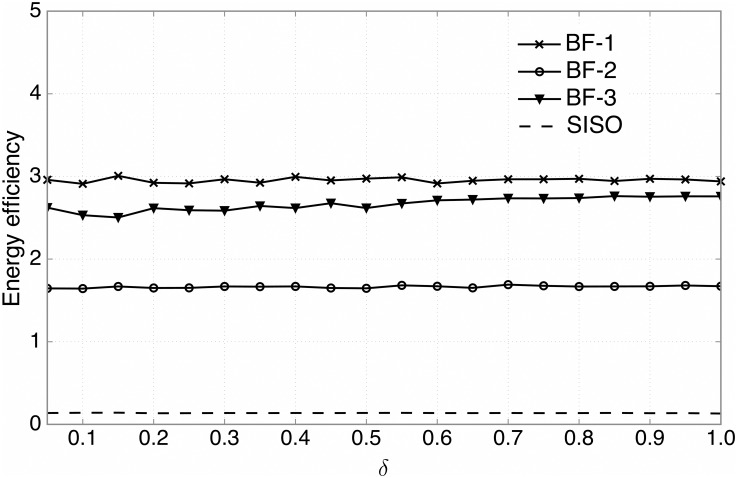
The energy efficiency performance. The energy efficiency versus the ratio of candidate SUs, *δ*, for three types of beamforming techniques (*N*_*t*_ = 2, *N*_*r*_ = 4) and the SISO case.

We now turn to evaluating the performance of energy efficiency defined as the ratio of the sum rate over the total transmit power consumed by all the SUs, since energy efficiency is an important performance metric in green communications [44, 45]. As illustrated in Fig 5, when *N*_*t*_ = 2 and *N*_*r*_ = 4, it is obvious that all three types of beamforming techniques outperform the SISO case for all range of *δ*, showing great improvements on the energy efficiency. As we scrutinize each type of the beamforming techniques, it is found that the BF-2 (the beamforming technique that minimizes the generated interference at the PBS) is less energy efficient, compared to the BF-1 (the beamforming technique that minimizes the required transmit power at the SU) and the BF-3 (the beamforming technique that maximizes the resulting SGINR). This is because the BF-2 design focuses only on the suppression of interference to the primary system, with no consideration of reducing the transmit power at the SUs.

### 4.2 Interference Temperature Suppression

**Fig 6:**
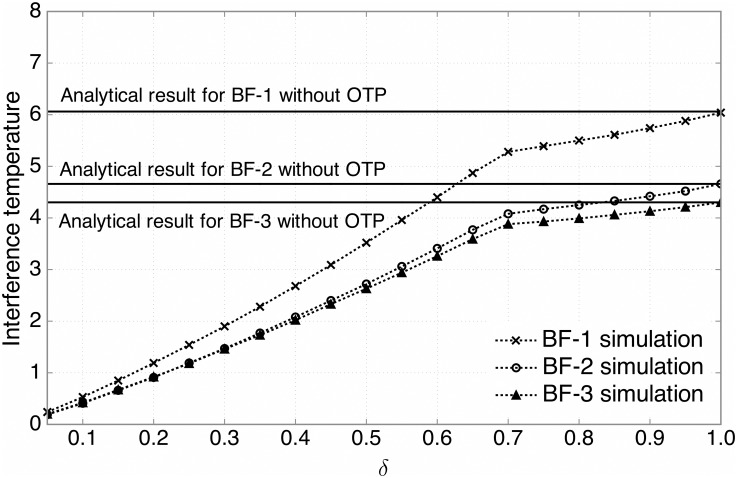
The interference temperature for three types of beamforming techniques. The average interference temperature versus the ratio of candidate SUs, *δ*, for three types of beamforming techniques (*N*_*t*_ = 4, *N*_*r*_ = 16). The analytical results with no OTP (*δ* = 1) in Eq (23) are also plotted for validation.

In this subsection, we measure the interference temperature suppression at the PBS, which is crucial to protect the successful reception of the primary system. The interference temperature here is given by $\sum_{i=1}^{K}e_{i}{∥G_{i}w_{i}∥}^{2}$, similarly as in [22, 25]. Fig 6 shows the average interference temperature at the PBS versus *δ*, where the antenna configuration is given by *N*_*t*_ = 4 and *N*_*r*_ = 16. It is observed that the BF-3 achieves the best performance among the three beamforming techniques in terms of interference temperature suppression, which thus validates the effectiveness of the SGINR-based beamforming. By maximizing the SGINR at each SU, we can not only enhance the desired signal power at the SBS but also reduce the interference at the PBS. It is also seen that performance of the BF-2 is quite comparable to that of the BF-3, while the BF-2 needs less complexity and a smaller amount of CSI. Moreover, for each beamforming technique, we find that as *δ* increases, a higher interference temperature at the PBS is generated since more SUs belong to the candidate group. As *δ* tends to 1, each simulation curve approaches the analytical result, corresponding to the case with no OTP (*δ* = 1) in Eq (24), which thus validates our analysis on the interference temperature in Section 3.1.

**Fig 7:**
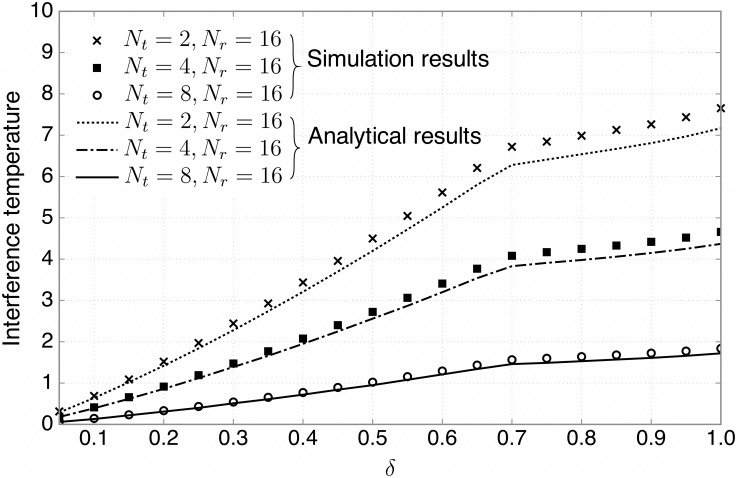
The interference temperature suppression. The average interference temperature versus the ratio of candidate SUs, *δ*, for various antenna configurations, where the BF-2 is employed. Both the numerical and analytical results are plotted.

**Fig 8:**
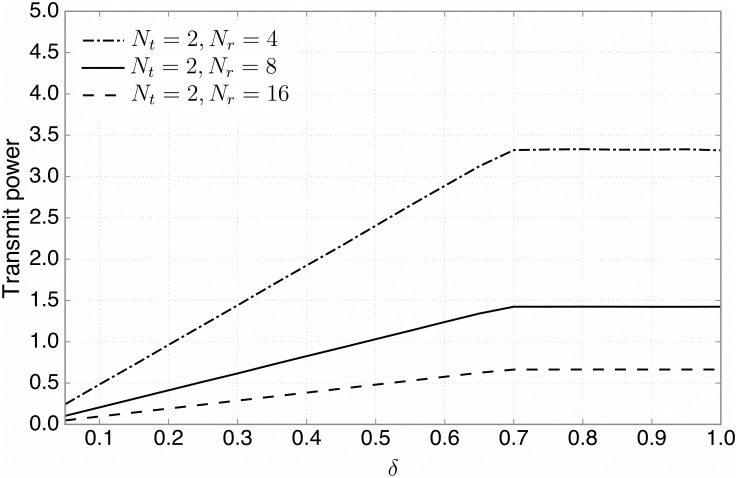
The transmit power performance. The total transmit power versus the ratio of candidate SUs, *δ*, for various antenna configurations, where the BF-2 is employed.

Now, we evaluate the interference temperature performance for various antenna configurations, where we focus on the BF-2 due to a good balance between the sum rate and interference suppression performance. We first vary the number of transmit antennas, while fixing the number of receive antennas, *N*_*r*_ (Fig 7), and then vice versa (Fig 8). As illustrated in Fig 7, when *N*_*r*_ = 16, the interference temperature can be significantly reduced by increasing *N*_*t*_, due to the fact that beamforming with more transmit antennas can suppress the interference at the PBS more effectively. In addition, it is observed that the numerical results are consistent with the analytical ones in Theorem 2 (Eq (23) for the BF-2 case). Fig 8 shows that when *N*_*t*_ = 2, increasing *N*_*r*_ results in less total transmit power. This is because by deploying more receive antennas at the SBS, the SUs can transmit with lower power since more receive diversity gain can be obtained.

### 4.3 Cognitive Transmission Capacity (CTC)

It is necessary to characterize a fundamental trade-off between the sum rate of the secondary system and the interference temperature at the PBS. To elaborate on this trade-off, we define a new performance metric, termed the CTC, which can also be used to find the optimal *δ*^⋆^ in terms of maximizing the net system throughput. The definition of CTC is based on the spectrum-sharing transmission capacity that was used to measure the area spectrum efficiency [46]. Specifically, the CTC in this work is given by *R*_sum_ ⋅ (1 − *P*_out_), where *R*_sum_ and *P*_out_ are the sum rate of the secondary system and the outage probability that the interference temperature at the PBS exceeds the constraint *E*_IT_, respectively. This metric can be interpreted as how much sum rate can be achieved by the secondary system without hindering the successful reception of the primary system.

**Fig 9:**
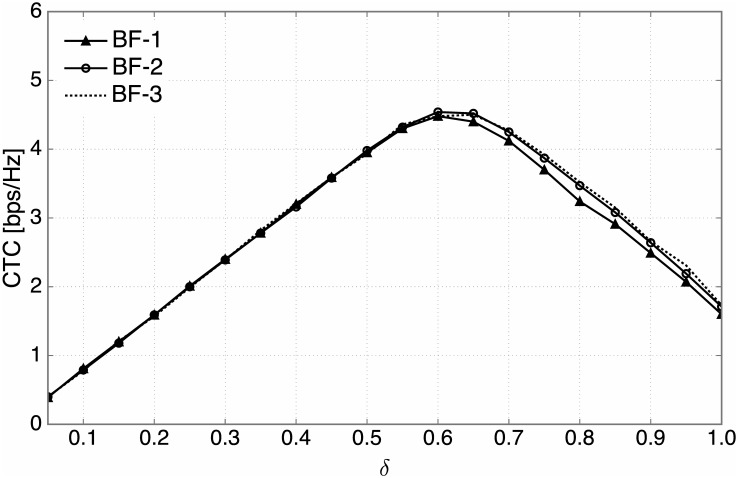
The CTC performance. The CTC versus the ratio of candidate SUs, *δ*, for three types of beamforming techniques (*N*_*t*_ = 4, *N*_*r*_ = 16).

As illustrated in Fig 9, for all three types of beamforming techniques, the CTC first tends to increase with *δ*, but starts to decrease when *δ* exceeds a certain value. Primary reasons of this phenomenon can be explained as follows. For small *δ*, since *P*_out_ is quite low, *R*_sum_ plays a more important role. Thus, the CTC increases with *δ* in this region. For sufficiently large *δ*, the increasing rate of *R*_sum_ becomes slow and *P*_out_ becomes dominant. As a result, the CTC begins to decrease with *δ*. Based on the observation that the resulting CTC curves are concave over *δ*, the optimal *δ*^⋆^ maximizing the CTC can be numerically found for each beamforming technique.

Table 2 shows the values of *δ*^⋆^ in terms of maximizing the CTC for the three types of beamforming techniques and various antenna configurations, which are obtained by numerical search. From this table, it is found that the optimal *δ*^⋆^ increases with *N*_*t*_. This is because using more transmit antennas enables to suppress the generating interference more effectively (as also shown in Fig 7), thus resulting in a lower outage probability. In consequence, more SUs can belong to the candidate group, leading to a superior CTC performance.

**Table 2:** Lookup table of *δ*^⋆^.

Antenna configuration	BF-1	BF-2	BF-3
*N*_*t*_ = 2, *N*_*r*_ = 16	0.40	0.45	0.45
*N*_*t*_ = 3, *N*_*r*_ = 16	0.45	0.50	0.60
*N*_*t*_ = 4, *N*_*r*_ = 16	0.55	0.65	0.70
*N*_*t*_ = 5, *N*_*r*_ = 16	0.55	0.75	0.70
*N*_*t*_ = 6, *N*_*r*_ = 16	0.60	0.70	0.80

Table 3 shows the optimal *η*^⋆^ (the threshold to control *δ* in the OTP) for the three types of beamforming techniques and various antenna configurations. In practice, the SBS obtains the optimal *η*^⋆^ by evaluating the CTC performance offline, and then broadcasts it over the network. In addition to the CTC metric, it is also possible to adjust *η* to achieve other QoS targets (e.g., using smaller *η* to greatly reduce the interference temperature at the PBS, at the cost of a relatively low sum rate in the secondary system).

**Table 3:** Lookup table of *η*^⋆^.

Antenna configuration	BF-1	BF-2	BF-3
*N*_*t*_ = 2, *N*_*r*_ = 16	14.67	11.00	12.46
*N*_*t*_ = 3, *N*_*r*_ = 16	15.15	8.76	11.86
*N*_*t*_ = 4, *N*_*r*_ = 16	16.18	7.58	11.29
*N*_*t*_ = 5, *N*_*r*_ = 16	16.17	6.49	10.31
*N*_*t*_ = 6, *N*_*r*_ = 16	16.69	5.01	10.33

### 4.4 Performance Evaluation Under a Standardized Model

**Fig 10:**
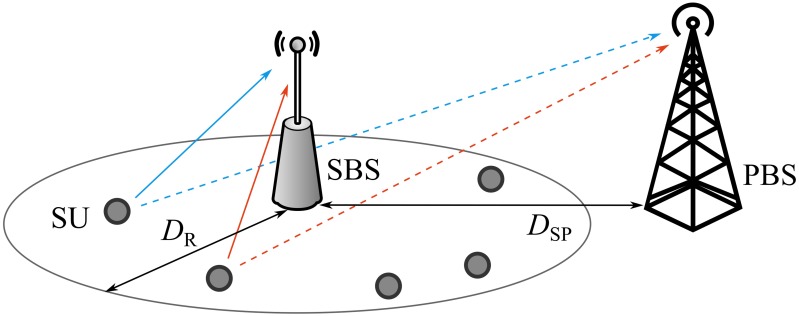
The system model assuming the practical setting. The SUs are uniformly distributed in the coverage of SBS with radius of *D*_R_. The PBS is placed *D*_SP_ away from the SBS.

In this subsection, we examine the system performance under a more realistic simulation environment, by taking into account both the random geometric distribution of the SUs and the path loss component of wireless links. According to practical wireless LAN applications, we adopt the following suitable propagation model for the IEEE 802.11n and 802.11ac standards [47]:

$$\begin{array}{cc} & PL\left(d\right)=20\log_{10}\left(d\right)+20\log_{10}\left(f\right)-147.5\;d\leq d_{\text{BP}}\\ & PL\left(d\right)=20\log_{10}\left(d_{\text{BP}}\right)+35\log_{10}\left(\frac{d}{d_{\text{BP}}}\right)+20\log_{10}\left(f\right)-147.5\;d>d_{\text{BP}}, \end{array}$$ (25)

where *d* [m] is the distance between one transmitter-receiver pair; *d*_BP_ [m] is the breakpoint; *PL*(*d*) is the path loss in dB scale; and *f* [Hz] is the frequency. As illustrated in Fig 10, on the coexistence with the primary system, we assume that the SUs are uniformly distributed in a two-dimension disc with a radius of *D*_R_ [m] and the SBS is located at the center, by referring to the simulation model in [48]. The PBS is located *D*_SP_ [m] away from the SBS.

The values of simulation parameters are summarized in Table 4.

**Table 4:** The simulation environment.

Parameter	Value
Breakpoint (*d*_BP_) for path loss	10m
Path loss exponent	2 for *d* ≤ *d*_BP_; 3.5 for *d* > *d*_BP_
Frequency band (*f*)	2.4GHz
Radius of SBS coverage (*D*_R_)	*D*_R_ ∈ {10, ⋯, 200}m
PBS to SBS distance (*D*_SP_)	*D*_SP_ ∈ {10, ⋯, 200}m
Number of SUs (*K*)	100
Decoding threshold (*ρ*)	0.0627
Antenna configuration	*N*_*t*_ = 2, *N*_*r*_ = 4
Beamforming type	BF-2

**Fig 11:**
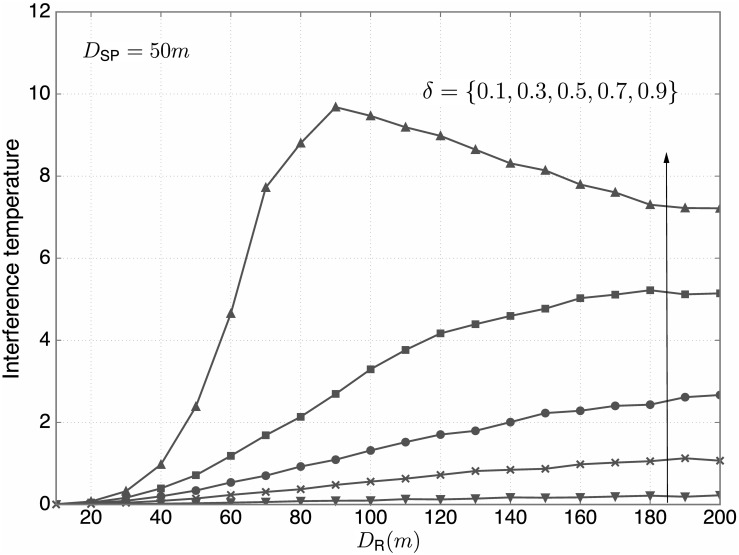
The interference temperature versus *D*_R_. For *D*_SP_ = 50m, the interference temperature versus *D*_R_ for various *δ* ∈ {0.1, 0.3, 0.5, 0.7, 0.9} is plotted.

Fig 11 shows the interference temperature versus *D*_R_ for *D*_SP_ = 50m and *δ* ∈ {0.1, 0.3, 0.5, 0.7, 0.9}. From the figure, the following observations are found:

- The interference temperature is monotonically increasing with *δ* for given *D*_R_, which is consistent with our analytical and numerical results based on the simulation environment with no path loss in Figs 7 and 8.
- For large *δ* (especially for *δ* = 0.9), the interference temperature first increases with *D*_R_ and then gets reduced. In this case, most of the SUs belong to the candidate group. If *D*_R_ < *D*_SP_, i.e., the PBS is located outside the SBS coverage, then all the generated interference is diminished by the path loss. If *D*_R_ ≥ *D*_SP_, i.e., the PBS is located inside the SBS coverage, then there may exist candidate SUs near the PBS and far away from the SBS, causing strong interference to the PBS. As *D*_R_ is very large, the resulting interference temperature tends to rather decrease due to more dispersed locations of SUs. More specifically, this comes from the fact that some SUs are far away from the PBS and the path loss greatly reduces the interference if these SUs transmit.

**Fig 12:**
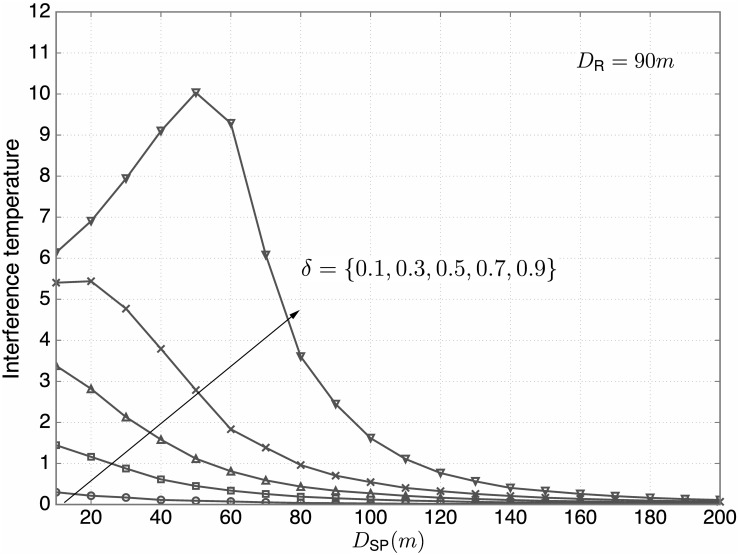
The interference temperature versus *D*_SP_. For *D*_R_ = 90m, the interference temperature versus *D*_SP_ for various *δ* ∈ {0.1, 0.3, 0.5, 0.7, 0.9} is plotted.

The distance between the PBS and SBS heavily affects the interference temperature suppression of the primary system due to the path loss component, which is verified through the following numerical results. Fig 12 shows the interference temperature versus *D*_SP_ for *D*_R_ = 90m (refer to [48]) and various *δ* ∈ {0.1, 0.3, 0.5, 0.7, 0.9}. From the figure, the following observations are found:

- The interference temperature is monotonically increasing with *δ* for given *D*_SP_.
- For small *δ* (e.g, *δ* ≤ 0.5), the interference temperature tends to increase with *D*_SP_ for all ranges of *D*_SP_.
- For large *δ* (especially for *δ* = 0.9), the interference temperature first increases with *D*_SP_ and then gets reduced. If the PBS is within the coverage of the SBS, then some SUs nearby the PBS will cause serious interference to the PBS. If the PBS is out of the coverage of the SBS, then the interference temperature decreases with *D*_SP_ since the impact on path loss becomes dominant.
- Based on the aforementioned observations, it is found that the OTP plays a crucial role on the interference temperature suppression if the PBS is within the coverage of the SBS, but is less important if the PBS is far away from the SBS. Thus, the distance between the PBS and SBS is important in determining *δ*.

**Fig 13:**
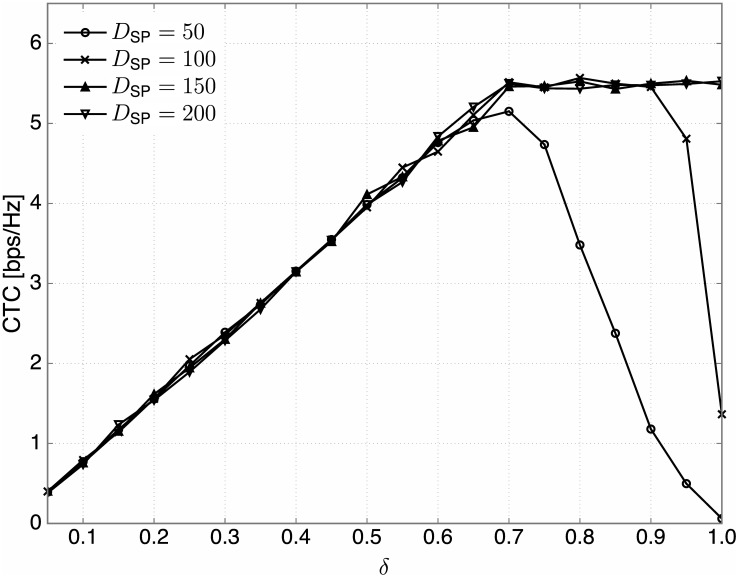
The CTC versus *δ* under the standardized model. For *D*_R_ = 90m, the CTC versus *δ* for various *D*_SP_ ∈ {50, 100, 150, 200}m is plotted.

From Figs 11 and 12, it is seen that proper values of *δ* are required to better control the interference temperature for different conditions on *D*_R_ and *D*_SP_. Recall that to find proper values of *δ*, we need to consider both the interference temperature at the PBS and the sum rate of the secondary system. Hence, performance on the CTC is also evaluated for our practical setting. Fig 13 illustrates the CTC versus *δ* for *D*_R_ = 90m and *D*_SP_ ∈ {50, 100, 150, 200}m. From the figure, the following observations are found:

- Since the CTC curves tends to be quasi-concave, it is possible to find the optimal values of *δ* (denoted by δ*) that maximize the CTC.
- The optimal values of *δ* for various *D*_SP_ are different from those found under the system model with no path loss component. This is because the distribution of fading coefficients depends heavily on the path loss. Thus, we need to numerically select δ* according to given *D*_SP_ and *D*_R_ in addition to other parameters.
- It is observed that the optimal δ* tends to increase with *D*_SP_. That is, more SUs may have an opportunity to transmit as the PBS is farther away from the SBS, since the interference is weakened by the path loss. When *D*_SP_ is sufficiently large (e.g., *D*_SP_ ≥ 150m), by virtue of the significant signal attenuation, almost all SUs are likely to belong to the candidate group to maximize the sum rate of the secondary system.

## 5 Concluding Remarks

For secondary random access in the MIMO CR network, we have proposed the OTP to opportunistically reduce the interference temperature at the PBS, by exploiting the multiuser diversity gain and efficiently combining three types of beamforming techniques and the modified DPA. The sum rate of the SUs and the interference temperature reduction at the PBS were evaluated through computer simulations. In particular, the average interference temperature was analytically shown and then was validated using simulations. It turned out that the beamforming designed in terms of maximizing the SGINR (the BF-3) achieves the best performance on both measurements. By using the CTC metric, we provided a novel framework for finding the optimal values of the OTP parameter that can be applied to practical MIMO CR networks operating in a decentralized fashion. In addition, the system performance was extensively examined under the realistic standardized model.
